# Supplementary material for: A Critical View of the Application of the APEX Software (Aqueous Photochemistry of Environmentally-Occurring Xenobiotics) to Predict Photoreaction Kinetics in Surface Freshwaters
Source: Molecules. 2019 Dec 18;25(1):9. doi: 10.3390/molecules25010009 (PMC7017383; doi:10.3390/molecules25010009)
Supplement: Supplementary file 1 [file molecules-25-00009-s001.zip › 3.2.4_gcc-4.4.0.zip!/3.2.4_gcc-4.4.0/3.2.4_gcc-4.4.0/doc/octave/html/interpreter/Saving-Data-on-Unexpected-Exits.html]

Saving Data on Unexpected Exits - Untitled


Up: Simple File I/O


---

##### 14.1.3.1 Saving Data on Unexpected Exits

If Octave for some reason exits unexpectedly it will by default save the
variables available in the workspace to a file in the current directory.
By default this file is named octave-core and can be loaded
into memory with the `load` command. While the default behavior
most often is reasonable it can be changed through the following
functions.

— Built-in Function: val = **crash\_dumps\_octave\_core** ()  
— Built-in Function: old\_val = **crash\_dumps\_octave\_core** (new\_val)  
> Query or set the internal variable that controls whether Octave tries
> to save all current variables to the file "octave-core" if it
> crashes or receives a hangup, terminate or similar signal.
>
>
>
> **See also:** octave\_core\_file\_limit, octave\_core\_file\_name, octave\_core\_file\_options.

— Built-in Function: val = **sighup\_dumps\_octave\_core** ()  
— Built-in Function: old\_val = **sighup\_dumps\_octave\_core** (new\_val)  
> Query or set the internal variable that controls whether Octave tries
> to save all current variables to the file "octave-core" if it receives
> a hangup signal.

— Built-in Function: val = **sigterm\_dumps\_octave\_core** ()  
— Built-in Function: old\_val = **sigterm\_dumps\_octave\_core** (new\_val)  
> Query or set the internal variable that controls whether Octave tries
> to save all current variables to the file "octave-core" if it receives
> a terminate signal.

— Built-in Function: val = **octave\_core\_file\_options** ()  
— Built-in Function: old\_val = **octave\_core\_file\_options** (new\_val)  
> Query or set the internal variable that specifies the options used for
> saving the workspace data if Octave aborts. The value of
> `octave_core_file_options` should follow the same format as the
> options for the `save` function. The default value is Octave's binary
> format.
>
>
>
> **See also:** crash\_dumps\_octave\_core, octave\_core\_file\_name, octave\_core\_file\_limit.

— Built-in Function: val = **octave\_core\_file\_limit** ()  
— Built-in Function: old\_val = **octave\_core\_file\_limit** (new\_val)  
> Query or set the internal variable that specifies the maximum amount
> of memory (in kilobytes) of the top-level workspace that Octave will
> attempt to save when writing data to the crash dump file (the name of
> the file is specified by octave\_core\_file\_name). If
> octave\_core\_file\_options flags specify a binary format,
> then octave\_core\_file\_limit will be approximately the maximum
> size of the file. If a text file format is used, then the file could
> be much larger than the limit. The default value is -1 (unlimited)
>
>
>
> **See also:** crash\_dumps\_octave\_core, octave\_core\_file\_name, octave\_core\_file\_options.

— Built-in Function: val = **octave\_core\_file\_name** ()  
— Built-in Function: old\_val = **octave\_core\_file\_name** (new\_val)  
> Query or set the internal variable that specifies the name of the file
> used for saving data from the top-level workspace if Octave aborts.
> The default value is `"octave-core"`
>
>
>
>
> **See also:** crash\_dumps\_octave\_core, octave\_core\_file\_name, octave\_core\_file\_options.
